# Monitoring the compaction of single DNA molecules in *Xenopus* egg extract in real time

**DOI:** 10.1073/pnas.2221309120

**Published:** 2023-03-14

**Authors:** Mingxuan Sun, Hossein Amiri, Alexander B. Tong, Keishi Shintomi, Tatsuya Hirano, Carlos Bustamante, Rebecca Heald

**Affiliations:** ^a^Department of Molecular and Cell Biology, University of California, Berkeley, CA 94720; ^b^Institute for Quantitative Biosciences (QB3), University of California, Berkeley, CA 94720; ^c^Chromosome Dynamics Laboratory, RIKEN, Wako 351-0198, Japan; ^d^Department of Physics, University of California, Berkeley, CA 94720; ^e^Department of Chemistry, University of California, Berkeley, CA 94720; ^f^HHMI, University of California, Berkeley, CA 94720; ^g^Kavli Nanosciences Institute, University of California, Berkeley, CA 94720

**Keywords:** DNA compaction, *Xenopus* egg extract, optical trap, chromatin assembly

## Abstract

DNA compaction is required for the condensation and resolution of chromosomes during mitosis, but the relative contribution of individual chromatin factors to this process is poorly understood. We developed a physiological, cell-free system using high-speed *Xenopus* egg extracts and optical tweezers to investigate real-time mitotic chromatin fiber formation and force-induced disassembly on single DNA molecules. Compared to interphase extract, which compacted DNA by ~60%, metaphase extract reduced DNA length by over 90%, reflecting differences in whole-chromosome morphology under these two conditions. Depletion of the core histone chaperone ASF1, which inhibits nucleosome assembly, decreased the final degree of metaphase fiber compaction by 29%, while depletion of linker histone H1 had a greater effect, reducing total compaction by 40%. Compared to controls, both depletions reduced the rate of compaction, led to more short periods of decompaction, and increased the speed of force-induced fiber disassembly. In contrast, depletion of condensin from metaphase extract strongly inhibited fiber assembly, resulting in transient compaction events that were rapidly reversed under high force. Altogether, these findings support a speculative model in which condensin plays the predominant role in mitotic DNA compaction, while core and linker histones act to reduce slippage during loop extrusion and modulate the degree of DNA compaction.

## Significance

Mechanisms underlying chromosome condensation are poorly understood. We combined *Xenopus* egg extracts with optical tweezers to examine the compaction of single DNA molecules under physiological conditions that support cell cycle–dependent chromosome assembly in vitro. Depletion of core histones, linker histones, or condensins had distinct effects that provide insight into their individual contributions to the condensed chromatin state. Whereas condensin is the major player driving compaction, histones act to stabilize the compacted DNA.

Chromatin compacts and stabilizes DNA, but must also assemble dynamically to facilitate a variety of cellular processes including replication and transcription during interphase of the cell cycle. During cell division, chromosomes further condense and resolve to facilitate their equal distribution to daughter cells. Approaches including reconstitution with purified components, high-resolution microscopy, and computational simulations have provided important insights into how mitotic chromosomes form (1, 2). Chromosome assembly is commonly viewed as a hierarchical process, with DNA wrapped around core histones (nucleosomes) as the fundamental unit of chromatin. Nucleosomes are stacked and assembled into thicker fibers by linker histones, and acted upon by a variety of ATPases including chromatin-remodeling enzymes, topoisomerases, and structural maintenance of chromosome (SMC) complexes that possess DNA loop-extruding activity such as condensin and cohesin. Remarkably, reconstitution of mitotic chromatid-like structures was shown to require only core histones and their associated chaperones, the strand-passing enzyme topoisomerase II (topo II), and condensin I (3). Mitotic phosphorylation of condensin I is also crucial for the individualization of mitotic chromosomes, and the current model is that condensins act together with topo II to form a series of nested chromatin loops extending radially from a central axis where they accumulate. However, core histones are not required for this process, since blocking nucleosome assembly in *Xenopus* egg extracts did not prevent the formation of mitotic chromosome structures, although they possessed more sparse and fragile DNA loops (4). Thus, a minimum number of factors contributing to chromosome condensation has been identified that can function independently of one another, at least in part, but their contributions at the scale of a single DNA molecule in a physiological context are poorly understood.

Here, we have established an assay to examine the dynamics of single DNA molecules at high temporal and spatial resolution using optical tweezers in cell extracts prepared from frog eggs. By depleting individual proteins and examining chromatin fiber assembly and force-induced disassembly, we show that condensins are the major driver of compaction, while histones act to stabilize the compacted DNA.

## Results

**Fig. 1.**
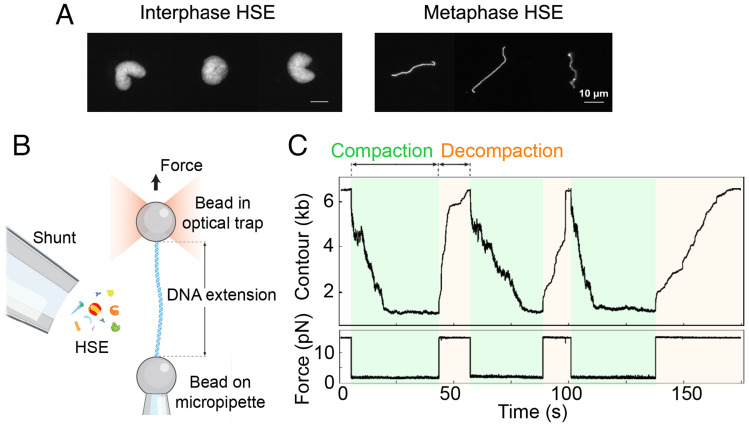
Assay using *Xenopus* egg extracts and optical tweezers to monitor compaction of a single DNA. (*A*) *Xenopus* sperm nuclei were added to high-speed extract (HSE) arrested in either interphase or metaphase. Sperm nuclei decondensed in interphase HSE to form rounded clusters of sperm chromosomes, whereas single mitotic chromosomes were visible in metaphase extracts. (*B*) Optical tweezer setup in which a linear DNA tether is coupled to two beads; one bead is attached to a micropipette and the other positioned in an optical trap. (*C*) Example trace showing DNA contour in base pairs and force exerted on the trapped bead over time. Alternating between low (1.5 pN) and high (15 pN) force after introduction of HSE results in rapid cycles of compaction and decompaction.

Crude egg extracts arrested in either interphase or metaphase were centrifuged at high speed to remove membranes and particles that would interfere with optical tweezer measurements. The morphology of sperm chromosomes added to the high-speed extracts (HSE) confirmed that the cell cycle state was maintained in these extracts, with decondensed chromatin and individual mitotic chromatids observed in interphase and metaphase, respectively (Fig. 1*A*). In the optical tweezers’ setup, a single linear 6.2 kb DNA fragment that was generated by PCR was tethered between two beads. One bead was maintained in a fixed position by a micropipette, while the second was held in an optical trap to monitor changes in force and DNA extension (Fig. 1*B*). After full extension of the DNA under high force (15 pN), HSE was introduced via a shunt. After an initial period of stabilization, DNA compaction was followed in real time by adjusting the position of the optical trap to maintain constant force at 1.5 pN. Compaction began immediately and was allowed to continue until the high extending force (15 pN) was reapplied, which led to unraveling of the DNA. With this assay, multiple cycles of compaction and decompaction of the DNA were followed by alternatively subjecting the molecule to 1.5 pN and 15 pN of force (Fig. 1*C*). Details of data analysis are provided in *SI Appendix*, Fig. S1, and the total number of compaction and decompaction cycles and traces analyzed under each condition is provided in *SI Appendix*, Table S1.

**Fig. 2.**
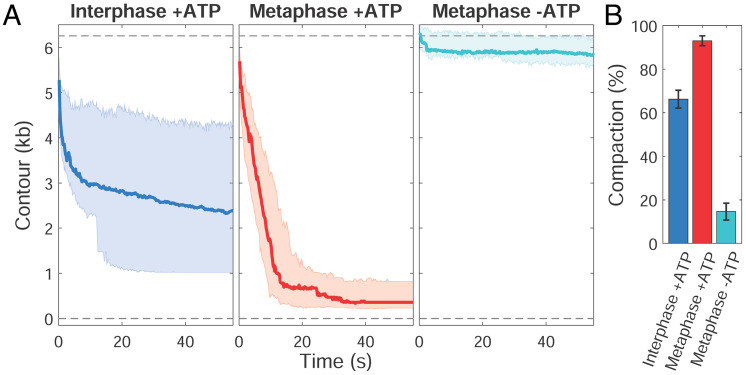
Cell cycle–dependent DNA compaction requires ATP. (*A*) DNA extension in base pairs over time following release from high force in interphase-arrested HSE with added ATP (blue), metaphase-arrested HSE with added ATP (red), and metaphase-arrested HSE without added ATP and treated with apyrase to degrade ATP (cyan). The thick line in each plot is the median trace and the bands indicate the upper and lower quartiles. Data are displayed at 5 Hz. Full-length (6,256 bp) and zero-length DNA contours are indicated with gray dashed lines. (*B*) Degree of compaction in each of the conditions in panel *A*. Individual traces are shown in *SI Appendix*, Fig. S3.

To examine cell cycle–dependent changes in single DNA compaction dynamics, we compared interphase and metaphase HSE (Fig. 2). Consistent with morphological differences at the scale of sperm chromosomes (Fig. 1*A*), we observed that DNA compaction was significantly greater in metaphase HSE. DNA compaction required energy, since treatment of HSE with apyrase to degrade ATP strongly inhibited it. The rapid time scale of compaction (< 30 s) demonstrated the high physiological activity of the HSE, which was shunted onto the DNA tether for only ~5 min before force on the bead was reduced and compaction began. The degree of compaction in interphase HSE was variable and may reflect the reported inefficiency in assembling high density, evenly spaced nucleosomes on naked DNA in egg extracts (5).

**Fig. 3.**
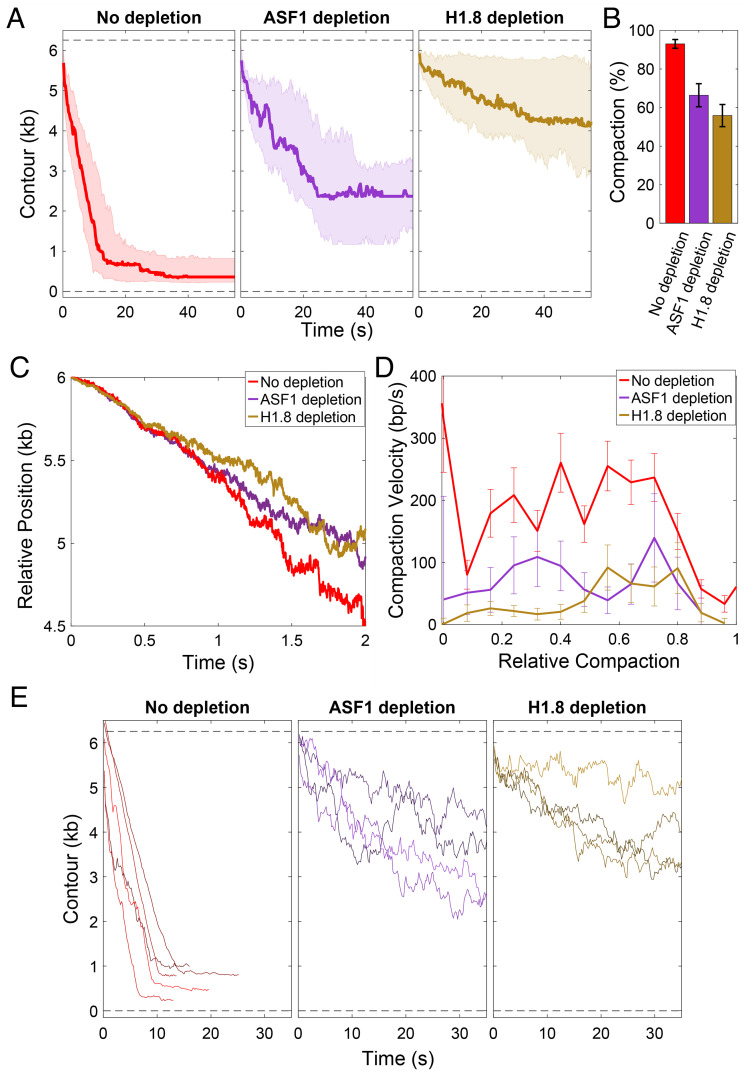
Core and linker histones contribute to single DNA compaction. (*A*) Compaction time course profiles upon no depletion (red), ASF1 depletion (purple), or linker histone H1.8 depletion (brown). The thick line in each plot is the median trace and the bands indicate the upper and lower quartiles. Data are displayed at 5 Hz. (*B*) Relative effects of ASF1 and H1.8 depletion on final compaction level. (*C*) Compaction dynamics in control and histone-depleted reactions. Curves show median trace of actively compacting fibers (*SI Appendix*). Fibers under depletion conditions condense more slowly after 1 s, indicating slippage. (*D*) Compaction velocities (mean ± SEM) as a function of relative compaction state. Rates of overall compaction are higher in control reactions, but do not vary significantly with the relative level of compaction in control or following core or linker histone depletion. *SI Appendix*, Fig. S3 (*E*) A subset of traces is shown to illustrate that more frequent upward movements are observed upon depletion of ASF1 or H1.8. See *SI Appendix*, Fig. S4*B* for quantification. Individual traces under each condition are shown in *SI Appendix*, Fig. S3.

We next evaluated the role of core and linker histones in DNA compaction by treating metaphase HSE with specific antibodies coupled to beads, which removed >95% of targeted proteins (*SI Appendix*, Fig. S2). Immunodepletion of the H3/H4 chaperone ASF1 was previously shown to prevent nucleosome assembly on naked DNA (4). This treatment decreased the level of compaction by 29% ± 6% (mean ± SEM), whereas depletion of the linker histone H1.8, the isoform found in egg extract, also called B4 and H1M (6, 7), had a more dramatic effect, decreasing overall compaction by 40% ± 6% (mean ± SEM) (Fig. 3 *A* and *B*, see *SI Appendix*, Fig. S3 for raw data). Both depletions slightly decreased compaction velocity in base pairs per second compared to controls (Fig. 3 *C* and *D*), at least in part due to the increased incidence of short periods of decompaction, indicating slippage (Fig. 3*E*), which accounted for ~30% of data points as opposed to less than 5% in the control (*SI Appendix*, Fig. S4*B*). Thus, core and linker histones both contribute to the dynamics and degree of DNA compaction.

**Fig. 4.**
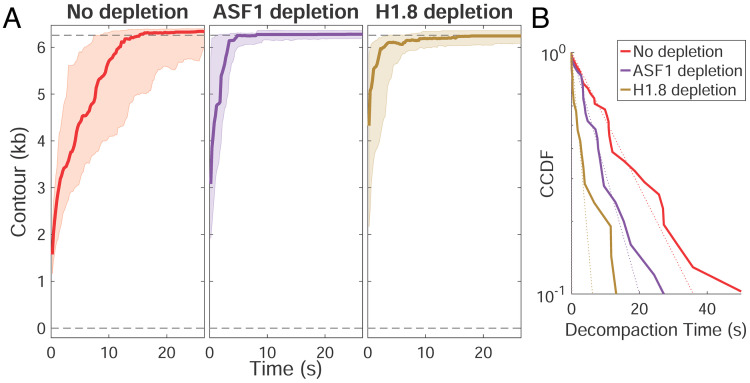
Core and linker histones contribute to the compacted state of the DNA fiber. (*A*) Decompaction time course profiles at high force in HSE with no depletion (red), ASF1 depletion (purple), or linker H1.8 depletion (brown). The thick line in each plot is the median trace and the bands indicate the upper and lower quartiles. Data are displayed at 5 Hz. (*B*) Complementary cumulative distribution function (CCDF) fitting to decompaction times show the extent to which ASF1 and H1.8 depletions reduce decompaction time. Individual traces for each condition are shown in *SI Appendix*, Fig. S3.

Consistent with a role for histones in stabilizing the metaphase compacted state, depletion of core or linker histones decreased the time required for decompaction when fiber extension was induced at high force (Fig. 4*A*). Fitting the decompaction time to a complementary cumulative distribution function (CCDF) revealed that histone H1.8 depletion reduced this time to a greater extent than core histone depletion (Fig. 4*B*). These observations further support the idea that linker histone H1.8 contributes more to DNA fiber compaction than core histones in metaphase HSE and suggest that binding of H1.8 to the nucleosome dyad axis (8), or directly to the DNA (4), significantly promotes the formation of the compacted fiber. Histone depletions did not significantly alter decompaction step size, which showed a median value of ~200 bp (or ~66 nm, *SI Appendix*, Fig. S4*B*), in agreement with previous studies on chromatin unraveling (9, 10).

**Fig. 5.**
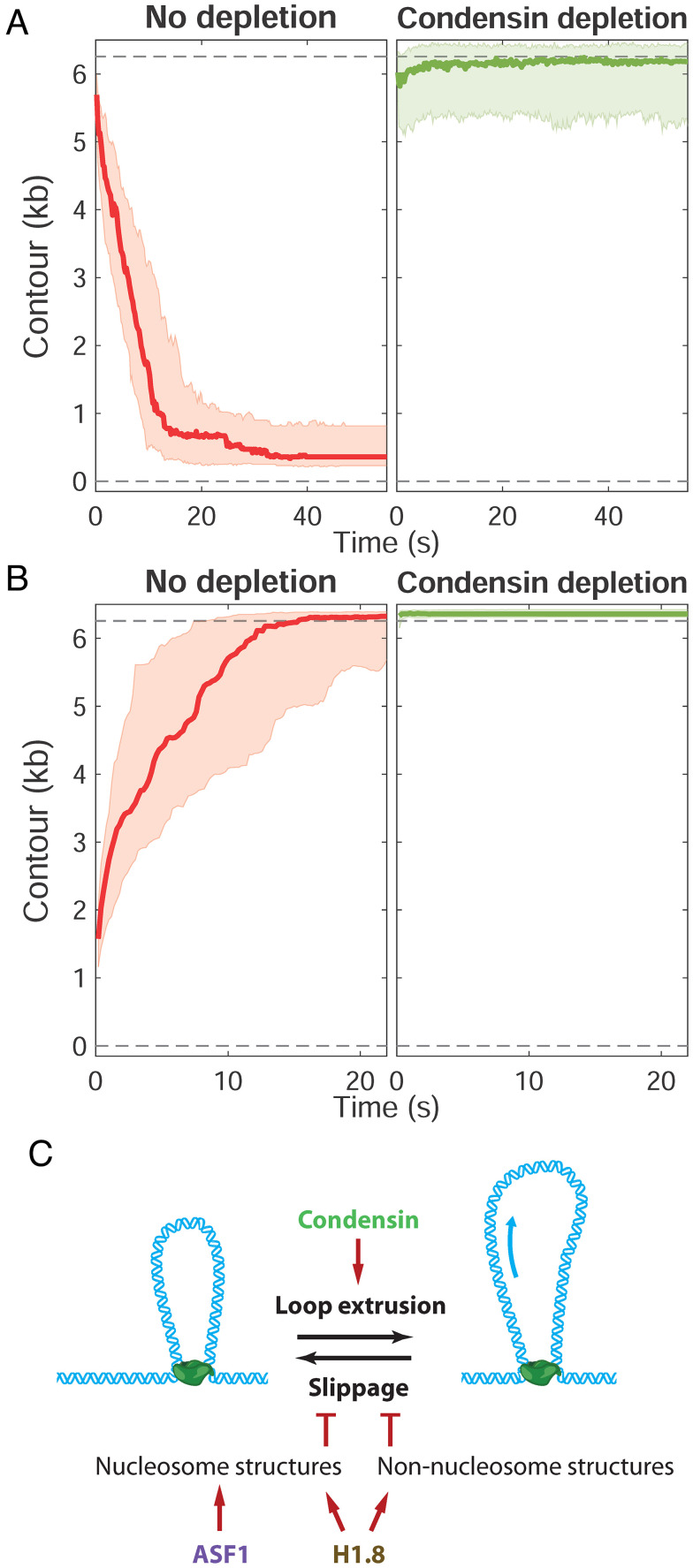
Effects of condensin depletion on compaction and decompaction dynamics. Time course profiles for compaction at low force (*A*) and decompaction at high force (*B*) in HSE with no depletion (red) and condensin depletion (green). The thick line in each plot is the median trace and the bands indicate the upper and lower quartiles. Data are displayed at 5 Hz. (*C*) Model in which condensin-extruded DNA loops are stabilized by formation of nucleosomes as well as nonnucleosomal H1.8-DNA interactions that inhibit condensin slippage.

Condensins are thought to be the main driver of mitotic chromosome condensation (11). Remarkably, upon depletion of both condensin I and II from metaphase HSE using antibodies to subunits of both complexes (*SI Appendix*, Fig. S2), DNA compaction was nearly abolished (Fig. 5*A*), an effect similar to ATP depletion (Fig. 2). Any compaction that occurred was rapidly reversed at high force (Fig. 5*B*), and although some meandering compaction traces were observed upon condensin depletion (*SI Appendix*, Fig. S3*A*), quantification of relative distance of compaction vs. slippage showed that unlike ASF1 or H1.8 depletion, condensin depletion resulted in neither consistent backward movements nor net compaction (*SI Appendix*, Fig. S4*B*). Thus, condensins are the major driver of DNA compaction in metaphase HSE, and histones and other DNA binding factors are not sufficient to generate a compacted fiber state under force.

## Discussion

*Xenopus laevis* egg extracts have provided a powerful system to examine DNA replication and repair in interphase as well as the morphology of individual mitotic chromosomes during metaphase (12). Here, taking advantage of this physiological system to monitor the behavior of a single DNA molecule enabled us to distinguish cell cycle–dependent differences in DNA compaction dynamics, as well as the effects of depleting key chromatin factors. The optical tweezers method employed here greatly improves spatial and temporal characterization of the assembly process compared to previous approaches in which single DNA molecules were combined with highly diluted extracts and required many minutes to compact (13). However, while the 6 kb DNA tether facilitated sensitive measurements, its short length and apparently sparse binding of core histones limit the relevance of this experimental system to whole chromosome condensation. Furthermore, the nature of the tweezer assay, in which a linear DNA is held under tension under assembly conditions, may constrain its topology in a way that alters the activity of DNA binding factors, for example by affecting supercoiling (14). Finally, depletion conditions could have secondary effects on other factors in the egg extract that could ultimately affect the compacted state.

A number of studies have shown that condensins are required for chromosome resolution, but not for the observed reduction in the volume of metaphase chromosomes (15). Instead, local compaction is thought to be driven by weak interactions between nucleosomes and other DNA-binding proteins that generate a condensed liquid-like phase (16). Our experiments support a key role for condensins in metaphase compaction at the scale of a single DNA, consistent with their ATP-dependent loop-extruding activity (17, 18). In current models, condensin II is thought to initially form large loops, with condensin I subsequently forming smaller nested loops within these (1, 19). Unlike somatic vertebrate cells, *Xenopus* egg extracts contain predominantly condensin I (20). Our observation that DNA compaction proceeds at a relatively constant rate over time in the presence or absence of histones (Fig. 3*D*) indicates that overall compaction is due to processive rather than cooperative activity of condensins. However, as noted above, the constant force exerted on the DNA under assembly conditions could potentially alter the activity of compaction factors, limiting our ability to interpret these results. Future experiments examining loop topologies and protein dynamics will be required to inform the mechanistic basis of condensin-driven DNA compaction in this assay.

Our finding that histone H1.8 depletion has a greater effect than core histone depletion on the extent of DNA compaction in metaphase HSE (Fig. 3) is unexpected, since full nucleosome occupancy would be predicted to reduce DNA length approximately sixfold, and linker histone H1 further reducing length an additional approximately twofold to threefold in a classical hierarchical compaction model (21). Interestingly, we observed that core and linker histones failed to decrease DNA contour length under low force in the absence of condensins (Fig. 5), indicating that if significant nucleosome assembly is occurring in metaphase HSE, it is happening only within condensin-generated DNA loops that are not subject to the extension force. Inefficient nucleosome assembly on naked DNA in extracts has been reported (5) and is also indicated by the limited degree of DNA compaction (~60%) in interphase HSE (Fig. 2). Although H1.8 binding to DNA was shown to be strongly enhanced by nucleosomes in egg extracts (22), we hypothesize that some fraction of linker histone H1.8 is binding to nonnucleosomal DNA, which has been shown to occur dynamically in *X. laevis* egg extracts (4, 23), and contributes to metaphase fiber compaction within condensin-generated loops either through short-range cross-linking or by promoting liquid–liquid phase separation (16). It was previously shown that H1.8 depletion from egg extract does not have a dramatic effect on sperm chromosome morphology in the absence of DNA replication (4, 7), which similarly is not occurring in our assay. However, increased fragility of unreplicated chromosomes in the absence of H1.8 was noted (4), consistent with our observation that DNA is less stably compacted under this condition. Notably, the H1.8 isoform found in *Xenopus* HSE differs significantly from somatic linker histones since its binding to DNA is highly dynamic and not subject to mitotic phosphorylation (24, 25). Although recently shown to bind to the nucleosome dyad (8), H1.8 was not included in the minimum set of factors required for mitotic chromosome reconstitution in egg extracts (3). Therefore, it remains to be determined exactly how H1.8 is functioning in this system and whether it plays an analogous role in vivo to facilitate rapid mitotic chromosome condensation.

One model consistent with our data is that core and linker histones prevent condensin slippage along the DNA, which has been observed under tension (26). The short periods of decompaction observed upon ASF1 or H1.8 depletion (*SI Appendix*, Fig. S4) also indicate that DNA slippage is common in the absence of histones. Altogether, we propose that condensin-dependent DNA loop extrusion is primarily responsible for DNA compaction in metaphase HSE and that core and linker histones stabilize the compacted state by inhibiting the reverse reaction (slippage), either by forming nucleosomes or through other modes of DNA binding by H1.8 (Fig. 5*C*). An interesting question is whether histones are sufficient to promote force-resistant DNA compaction in interphase HSE, or if other enzymes such as chromatin-remodeling complexes or the loop-extruding enzyme cohesin is required. Since the extract-tweezers system described in this study is highly amenable to biochemical manipulation, different factors can be either added or depleted under physiological cellular conditions. Future work applying this approach will allow evaluation of the individual roles of condensin I and condensin II, effects of depleting other nonhistone factors in chromatin fiber dynamics, including topo II, cohesin, and the chromokinesin Kif4A (2), as well as the interplay among different factors that influence their association with DNA (27, 28).

## Materials and Methods

### Preparation of High-Speed Extract (HSE) from *X. laevis* Eggs.

*X. laevis* frogs obtained from Nasco or the National *Xenopus* Resource (Woods Hole, MA) were used and maintained in accordance with standards established by the UC Berkeley Animal Care and Use Committee and approved in our Animal Use Protocol. Frogs were housed in a recirculating tank system with regularly monitored temperature and water quality (pH, conductivity, and nitrate/nitrite levels).

High-speed egg extracts (HSEs) were prepared and tested as described (29). Unless otherwise stated, all chemicals were purchased from Sigma-Aldrich. Briefly, freshly laid, metaphase II-arrested eggs were collected, dejellied, packed, and crushed by centrifugation. The crude, metaphase-arrested cytoplasm was collected from the middle of the tube with a syringe and 18G needle, and then supplemented with 10 µg/mL leupeptin, pepstatin, and chymostatin; 20 µM cytochalasin B; and 1/40 volume of energy mix (150 mM creatine phosphate, 20 mM ATP, 2 mM EGTA, 20 mM MgCl_2_). To prepare interphase-arrested crude extract, 0.4 mM CalCl_2_ and 50 µg/mL cycloheximide were added to a portion of the extract and incubated for 90 min at 20 °C. To obtain HSE, the crude extract (mitotic or interphase) was clarified by centrifugation in a TLS-55 rotor at ~200,000 g for 2 h at 4 °C. The middle layer was collected and centrifuged at 200,000 g for 30 min at 4 °C. Finally, the supernatant was flash-frozen and stored at −80 °C. To test HSE chromatid condensation activity, an equal volume of EB buffer (80 mM β-glycerophosphate, 15 mM MgCl_2_, 20 mM EGTA, pH 7.3) was added along with 1/20 volume energy mix, and the reaction was kept on ice. After addition of sperm nuclei (~500 to 1,000 nuclei/µL), compaction was monitored under oxygen-scavenging conditions, with 10 nM Sytox Green DNA dye, on an epifluorescence microscope setup equipped with FITC-channel filters and a 60× objective.

### Immunodepletion and Apyrase Treatment of HSE.

Immunodepletions were performed by incubating antibodies coupled to beads with HSE. For H1.8, 50 μg affinity-purified anti-H1M antibody was coupled to 200 μL protein A Dynabeads (Thermo Fisher) as described (7). For ASF1, 50 μL antiserum was coupled to 50 μL protein A sepharose beads (GE Healthcare) as described (4). For condensin, 10 μg each of affinity-purified antibodies against CAP-E, CAP-G, and CAP-D3 was coupled to 100 μL protein A Dynabeads (20). Antibody-coated beads were washed three to five times in XB buffer (10 mM HEPES pH 7.7, 100 mM KCl, 1 mM MgCl_2_, 0.1 mM CaCl_2_, 50 mM sucrose) for H1.8, and TBS-TX buffer (20 mM Tris-HCl pH7.5, 150 mM NaCl, 0.1% Triton X-100) for ASF1 and condensin. Beads were then incubated with 100 μL HSE on ice for 30 to 45 min with intermittent gentle agitation, and the depleted supernatant was carefully recovered away from the beads. In each case, two rounds of depletion were performed, as well as a mock depletion using nonimmunized rabbit IgG antibodies. Western blotting verified the degree of depletion (typically >~95%) (*SI Appendix*, Fig. S2). ATP was depleted by adding 0.02 U/μL apyrase (A6410; Sigma-Aldrich) to the HSE followed by 15 min incubation at 25 °C.

### Optical Tweezers Assays.

The 6.2 kb DNA tether was amplified by PCR from lambda phage genomic DNA using a 5′-biotinylated primer (5’-biotin-gaacagacagaggacgccg), and a 5′-digoxigenin labeled primer (5’-dig-ggccatgttgttgctgtatgc) (IDT). Control or depleted frozen 100 μL HSE aliquots were thawed, an equal volume of EB buffer was added, and the mixture was supplemented with 1/20 volume energy mix. The reaction was kept on ice and clarified by centrifugation at 12,000 g for 15 min at 4 °C. Force spectroscopy (optical tweezers) experiments were performed with dual-beam single-trap instruments equipped with force feedback using fluidics chambers constructed in house (30, 31). The flow cell was washed with PBS and 0.5 mg/mL bovine serum albumin to prevent nonspecific binding. The DNA was deposited on 2 µm polystyrene beads (Spherotech) coated with anti-dig antibody. DNA tethers were formed between an anti-dig-coated bead held in the optical trap and a streptavidin-coated bead that was held in place by a suction micropipette. Stable single tethers were verified by their force-vs.-extension fit to the worm-like chain model and by their overstretching behavior. Approximately 5 µL extract was flown through the chamber via a shunt for ~5 min while the tether was maintained at a high (15 pN) constant force. The constant force was then changed to ~1.5 pN to allow DNA compaction, and again to 15 pN for decompaction, with several cycles performed for each tether. The chamber was rinsed extensively with XB buffer before a new bead pair was used to form another tether. Each extract was used within ~3 h of thawing.

### Data Analysis.

The traces were transformed from units of tether extension (distance between the beads) to DNA contour (length of the uncompacted DNA between the beads) via fitting each trace’s force-extension curve to the extensible worm-like chain model (*SI Appendix*, Fig. S1). The traces yielded an average persistence length of 34 nm and stretch modulus of 1,300 pN. The unit change was done because extension is force dependent, the 6,256 bp tether at 1 pN has an estimated extension of 1,800 nm, but 2,100 nm at 15 pN. However, in contour length space, both values are 6,256 bp. In addition, the fitting sets a zero point for the length, since there is no reference point between the micropipette and the trap in this optical tweezers setup.

Degree of compaction is calculated as the end point of the compaction trace divided by the total length of the tether (6,256 bp). Velocity is calculated by a Savitzky–Golay differentiating filter of degree 1, width 0.5 s, to obtain filtered velocities at each point. See *SI Appendix* for more details.

## Supplementary Material

Appendix 01 (PDF)

## Data Availability

All study data are included in the article and/or *SI Appendix*. Raw data are deposited in Dryad (doi: 10.6078/D1JD9S).
