# Supplementary material for: Monitoring the compaction of single DNA molecules in Xenopus egg extract in real time
Source: Proc Natl Acad Sci U S A. 2023 Mar 14;120(12):e2221309120. doi: 10.1073/pnas.2221309120 (PMC10041109; doi:10.1073/pnas.2221309120)
Supplement: Supplementary file 1 — Appendix 01 (PDF) [file pnas.2221309120.sapp.pdf]

PNAS

Supporting Information for

Monitoring the condensation of single DNA molecules in *Xenopus* egg extract in real time

Mingxuan Sun, Hossein Amiri, Alexander B. Tong, Keishi Shintomi, Tatsuya Hirano, Carlos Bustamante, and Rebecca Heald

Corresponding author: Rebecca Heald
Email: bheald@berkeley.edu

This PDF file includes:

Supporting text
Figures S1 to S4
Table S1

Supporting Information Text

Data Analysis

Force and extension data were obtained from each DNA tether at 1 kHz. To convert DNA extension in nanometers to contour length (in base pairs), a pulling curve was first obtained and the stretching behavior of the tether was fit to an extensible worm-like chain model (Figure S1). The contour length is extracted from this fit knowing that the starting DNA is 6256 bp long and allowing for an extension offset accounting for bead size variability (Figure S1A). Note that, in contour length space, changes in force do not register as changes in contour length in base pairs, as the amount of DNA that makes up the tether is unchanged (Figure S1B). However, when extracts are introduced via the shunt, we do see a shortening of the measured contour length, as portions of the DNA tether are 'sequestered' during compaction which effectively shortens the total length of the DNA tether.

Traces were divided up into compaction cycles: periods when the tether was held at low force to allow the tether to compact, and decompaction cycles: periods following compaction cycles when the tether is then held at high force and allowed to extend. The compaction and decompaction cycles were combined and plotted by $25^{th}/50^{th}/75^{th}$ quartile (Figures 2A, 3A, 4A, 5A, 5B, S3). The extent of compaction was taken from the final length of the DNA at the end of the compaction cycle and dividing by the DNA length (6256 bp) (Figures 2B, 3B). The compaction velocity was calculated by filtering the traces with a Savitsky-Golay differentiating filter of width 500 ms, and order 1. This calculates the instantaneous velocity at each point by fitting a line to the points in the 500 ms window. The velocities can be used to identify regions that are compacting (negative velocity), paused (zero velocity), and decompacting/slipping (positive velocity) by fitting the velocity-time trace to a three-state hidden markov model. Regions of compaction were extracted, shifted to start at 6000 bp (arbitrary), and averaged together to obtain Figure 3C. The average velocity in every 500 bp position window (e.g., the average velocity of traces that are at 5500-6000 bp) was taken for Figure 3D. To quantify the amount of slipping during compaction for each condition, the trajectories were downsampled to 1Hz and the point-by-point difference was taken. Negative values (compaction) and positive values (extension) were summed separately, and the ratio of |compaction| / (|compaction| + extension) was taken for each trace and plotted as a beeswarm for Figure S4A.

Decompaction time was measured by taking the time it took from the start of the decompaction cycle to crossing y = 6000 bp (96% of the expected fully-extended length). These times were fit to a single exponential for Figure 4B. Step-finding for decompaction traces was done by finding peaks in the trace's residence time histogram. In order to make peak detection simpler, the histogram was smoothed by a Gaussian window with a width of 20bp (equal to the noise of the data) that reflects the position uncertainty. The distance between peaks was taken as the step size, the histogram of which is plotted in Figure S4B.

All analysis was done in Matlab R2016a. Code is available at doi:10.5281/zenodo.7687027.

Supporting Figures and Table

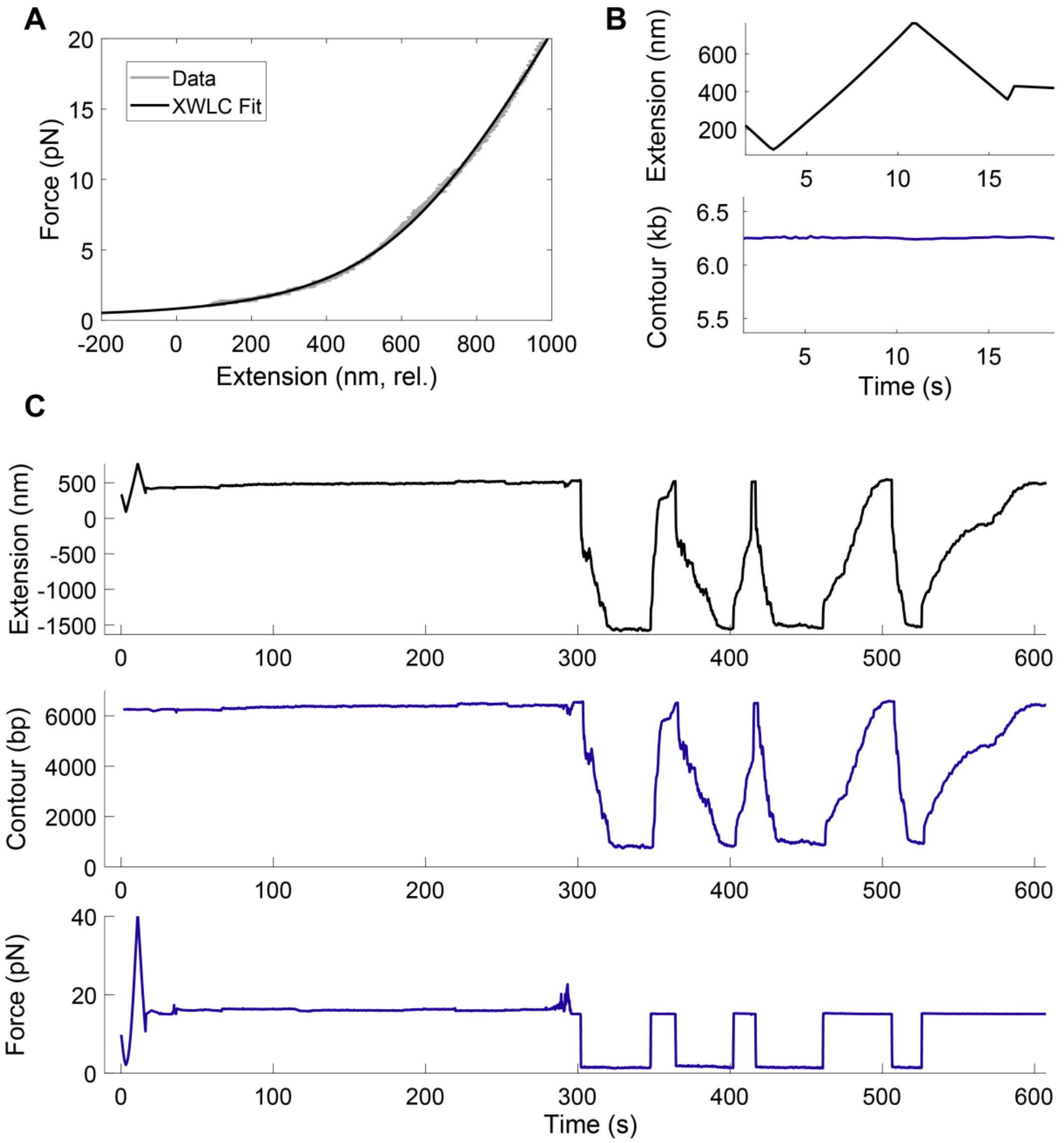

Fig. S1. Optical tweezers DNA contour length analysis. (A) Force-extension curve of the ~6.2 Kb DNA tether in gray, and an Extensible Worm Like Chain (XWLC) fit in black. (B) The contour length calculated based on the XWLC parameters remains flat if the DNA is simply pulled or relaxed. (C) Example trace showing raw data of DNA extension length over time in the top panel converted to contour length in base pairs in the middle panel. The lower panel shows force exerted on the trapped bead.

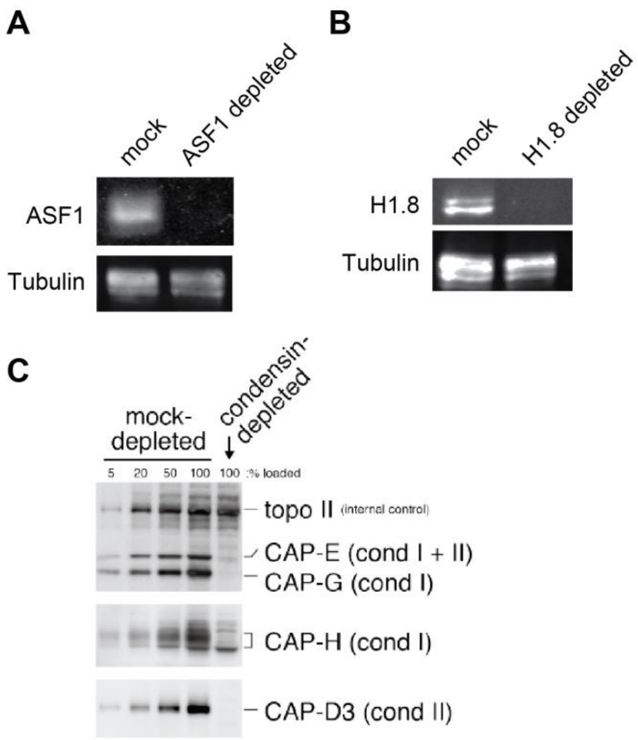

Fig. S2. Western blots of ASF1, histone H1.8, and condensin depletions.

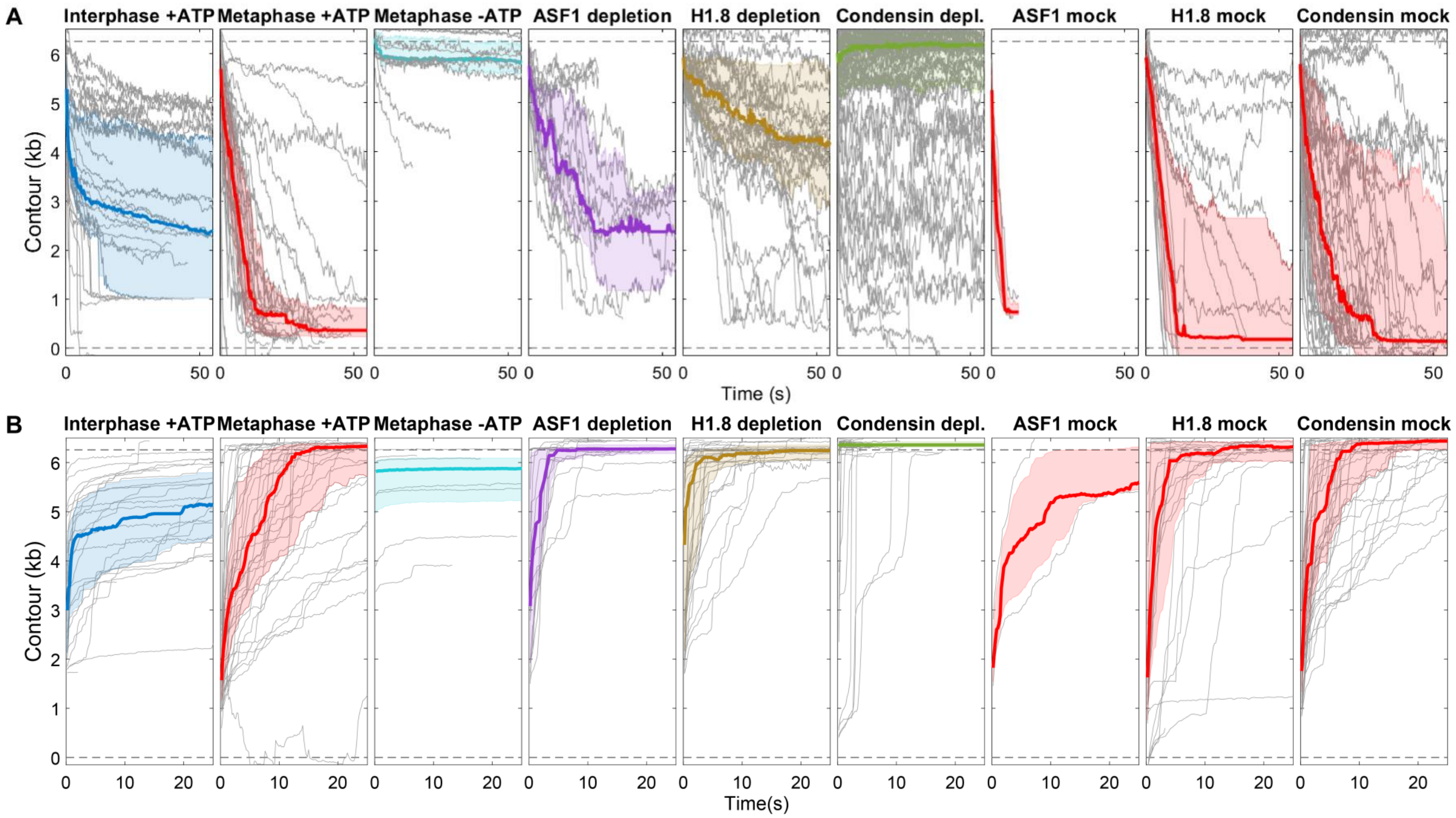

Fig. S3. Individual experiment traces for all conditions tested in this study. (A) Compaction traces showing DNA contour length over time following release from high force. (B) Decompaction traces for the same conditions following return to high force. Individual traces are in gray. The median trace and the quartile bands are colored. The dashed line at 6256 bp corresponds to the expected contour length of full-length naked DNA.

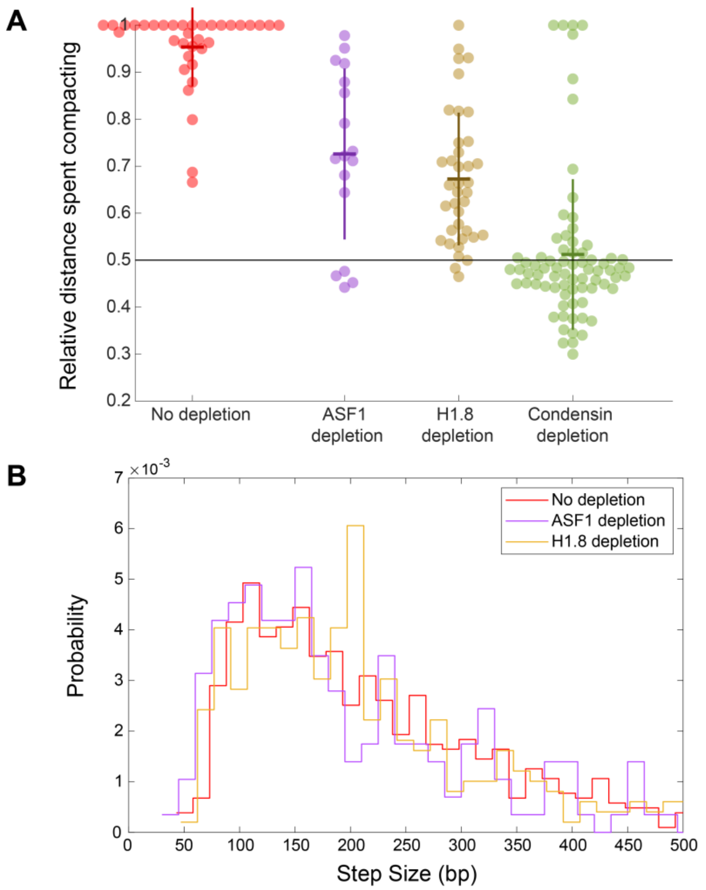

Fig S4. Frequency of short decompaction phases and quantification of step sizes. (A) Quantification of compaction versus slippage. The ratio between the distance moved downwards (compaction) versus total movement (upwards + downwards) was calculated for each compaction trace. With no depletion, 95% of movement is compaction, compared to 72% and 67% for ASF1 and H1.8 depletions, respectively. The black line at 0.5 indicates the expected ratio with no net compaction, as is the case for most condensin depletion traces. Each point represents a single trace with the mean and standard deviation indicated by solid lines. (B) Step size distribution of decompaction traces. The decompaction step sizes show a similar distribution across the different conditions tested. The median step size is ~200 bp and the mean is ~250bp.

Table S1. Summary of experiments analyzed. For each single DNA tether, multiple compaction (low force) and decompaction (high force) traces were collected.

Condition	Interphase	Metaphase	Metaphase – ATP	ASF1 depletion	H1.8 depletion	Condensin depletion	ASF1 mock	H1.8 mock	Condensin mock
Number of compaction traces	30	41	12	17	39	77	4	22	51
Number of decompaction traces	33	47	14	20	42	60	5	28	58
Number of DNA tethers	8	6	4	4	6	16	2	7	11
